# Dibrom­ido(4′-phenyl-2,2′:6′,2′′-terpyrid­yl)copper(II) hemihydrate

**DOI:** 10.1107/S1600536810010585

**Published:** 2010-03-27

**Authors:** Zhen Ma, Chunyan Bi, Guangyou Ran, Zhang Wu, Baoqing Liu, Miao Hu, Yanpeng Xing

**Affiliations:** a School of Chemistry and Chemical Engineering, Guangxi University, Guangxi 530004, People’s Republic of China; b Fujian Institute of Research on the Structure of Matter, Fuzhou, Fujian, 350002, People’s Republic of China

## Abstract

The title Cu^II^ complex, [CuBr_2_(C_21_H_15_N_3_)]·0.5H_2_O, was obtained by the hydro­thermal reaction of copper(II) bromide, 4′-phenyl-2,2′:6′,2′′-terpyridyl (4′-Ph-terpy or *L*) and sodium citrate in water in 31% yield. There are two unique complex mol­ecules and a water mol­ecule in the asymmetric unit. The Cu^II^ cation is ligated by three N atoms of *L* and two bromide anions, forming an irregular CuN_3_Br_2_ polyhedron with a distorted square-pyramidal coordination geometry. In the crystal structure, O—H⋯Br hydrogen bonds link the mol­ecules in a three-dimensional network.

## Related literature

For the structures, properties and applications of *MLX*_2_ compounds (*M* = transition metal, *L* = terpyridine, *X* = halogen), see: Arriortua *et al.* (1988 ▶); Bugarcic *et al.* (2004 ▶); Kickelbick *et al.* (2002 ▶); Koo *et al.* (2003 ▶); Ma *et al.* (2009 ▶); Yam *et al.* (2003 ▶). For the preparation of the ligand, see Constable *et al.* (1990 ▶).  
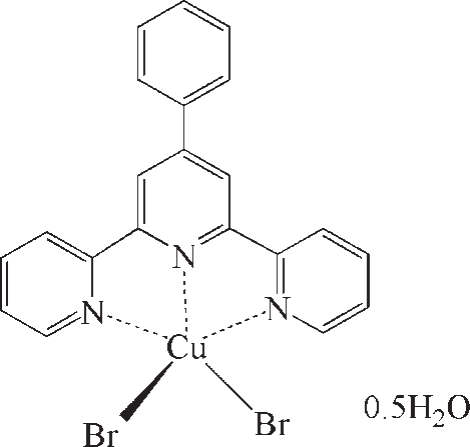


## Experimental

### 

#### Crystal data

[CuBr_2_(C_21_H_15_N_3_)]·0.5H_2_O
*M* *_r_* = 541.73
Triclinic, 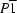

*a* = 10.1369 (16) Å
*b* = 10.8131 (17) Å
*c* = 18.688 (3) Å
α = 73.629 (3)°
β = 77.088 (4)°
γ = 87.567 (5)°
*V* = 1915.2 (5) Å^3^
*Z* = 4
Mo *K*α radiation
μ = 5.33 mm^−1^
*T* = 130 K
0.35 × 0.25 × 0.10 mm

#### Data collection

Rigaku Mercury CCD diffractometer
Absorption correction: multi-scan (*CrystalClear*; Rigaku, 2002 ▶) *T*_min_ = 0.543, *T*_max_ = 1.000
14948 measured reflections
8650 independent reflections
7545 reflections with *I* > 2σ(*I*)
*R*_int_ = 0.021

#### Refinement

*R*[*F* ^2^ > 2σ(*F* ^2^)] = 0.032
*wR*(*F* ^2^) = 0.079
*S* = 1.02
8650 reflections
496 parameters
2 restraints
H-atom parameters constrained
Δρ_max_ = 0.96 e Å^−3^
Δρ_min_ = −0.55 e Å^−3^

### 

Data collection: *CrystalClear* (Rigaku, 2002 ▶); cell refinement: *CrystalClear*; data reduction: *CrystalClear*; program(s) used to solve structure: *SHELXS97* (Sheldrick, 2008 ▶); program(s) used to refine structure: *SHELXL97* (Sheldrick, 2008 ▶); molecular graphics: *SHELXTL/PC* (Sheldrick, 2008 ▶); software used to prepare material for publication: *SHELXL97*.

## Supplementary Material

Crystal structure: contains datablocks I, global. DOI: 10.1107/S1600536810010585/sj2741sup1.cif

Structure factors: contains datablocks I. DOI: 10.1107/S1600536810010585/sj2741Isup2.hkl

Additional supplementary materials:  crystallographic information; 3D view; checkCIF report

## Figures and Tables

**Table 1:** Hydrogen-bond geometry (Å, °)

*D*—H⋯*A*	*D*—H	H⋯*A*	*D*⋯*A*	*D*—H⋯*A*
O1—H01⋯Br1^i^	0.85	2.89	3.520 (2)	133
O1—H01⋯Br2^i^	0.85	2.85	3.549 (2)	141
O1—H02⋯Br3^ii^	0.86	2.59	3.414 (2)	160

Symmetry codes: (i) 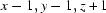; (ii) 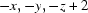.

**Fig. 1.**
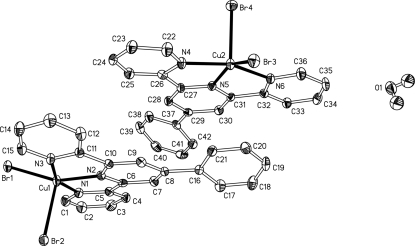
A view of the title complex, showing the atom labeling scheme with 50 % probability displacement ellipsoids. H atoms have been omitted for clarity. Cu-ligand bonds are indicated by full lines.

**Fig. 2.**
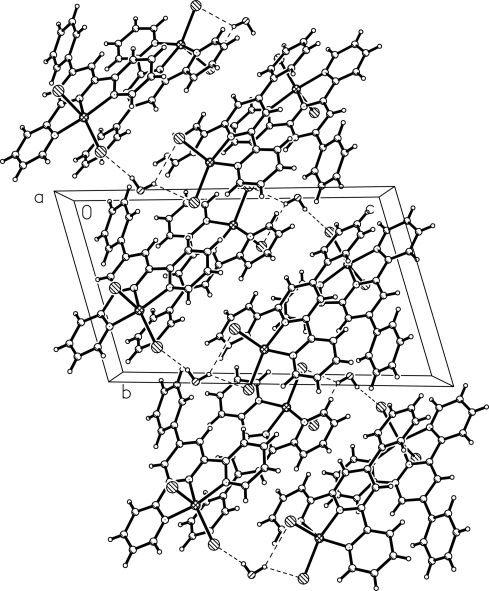
A view of the crystal packing along the a axis, showing the hydrogen bonding scheme. Hydrogen bonds are represented by dashed lines.

## supplementary crystallographic information

### Comment

The design and synthesis of photo-luminescent metal coordination compounds bearing terpy ligands have attracted a considerable interest. Examples include various terpy complexes of Pd(II), Pt(II), Zn(II) and Ag(I), (Bugarcic *et al.*, 2004; Ma *et al.*, 2009; Yam *et al.*, 2003). Despite a preparative route which involves the presence of sodium citrate in the hydrothermal reaction mixture, the single crystal structure of the title complex exhibits a neutral mononuclear unit with a copper(II) cation, one 4'-Ph-terpy ligand and two bromide ions in its coordination sphere. There is no evidence of coordination by the citrate anion to the central metal ion.

There are two independent complex molecules of [CuBr_2_(C_21_H_15_N_3_)]_2_.H_2_O in the asymmetric unit of the triclinic unit cell (Figure 1). Each copper (II) cation is coordinated by the three nitrogen atoms of the 4'-phenyl-2,2':6',2''-terpyridyl ligand, L, and two bromide anions, forming an irregular distorted square pyramidal CuN_3_Br_2_ polyhedron. In both unique molecules, the two bromide ions occupy the apical [Br(2) and Br(4)] and equatorial [Br(1) and Br(3)] positions. The other three basal coordination positions are occupied by the three nitrogen atoms of L. This geometry is confirmed by the angles between the ligand donor atoms in the equatorial plane of the square pyramid (Table 1). The angles between the two apical bromide ions and the three terpy nitrogen atoms are in the range 95.37 - 101.39 ° for one molecule and 94.30 - 102.29 ° for the other. The terpyridyl molecules in the two compounds are nearly planar (with RMS deviations 0.1446 for one compound and 0.0292 for the other). The phenyl rings of the terpy ligands are twisted to make angles of 17.1 (1) and 25.3 (2) ° respectively with the CuN_3_ coordination planes. Similar irregular CuN_3_Br_2_ polyhedra have been found in other copper(II) complexes (Arriortua *et al.*, 1988; Kickelbick *et al.*, 2002; Koo *et al.*, 2003). A lattice water is contained in the asymmetric unit, which originates from water used in the synthesis and is also involved in weak Cu—Br···H_2_O hydrogen bonding with the neighboring bromide ligands (Br(1), Br(2)) and Br(3)), respectively.

### Experimental

Free L was prepared by a reported procedure. (Constable *et al.*, 1990). The title compound was synthesized by reaction of copper(II) bromide, sodium citrate and L in hydrothermal conditions as follows: A mixture of CuBr_2_.4H_2_O (0.044 g, 0.14 mmol), L (0.040 g, 0.071 mmol), sodium citrate (Na_3_C_6_H_5_O_7_.2H_2_O) (0.021 g, 0.071 mmol), and distilled water (20 ml) was sealed in a 25 ml stainless steel reactor with a Teflon liner and heated at 110 °C for 3 days. Blue crystals of 1, suitable for X-ray characterization, were isolated by mechanical separation from a mixture which included an unidentified powder. The yield of 1 was 31 % based on the ligand.

### Refinement

Hydrogen atoms bonded to the ligands were positioned geometrically and refined using a riding model with C—H = 0.93 - 0.97 Å and with Uiso(H) = 1.2 times Ueq(C).These hydrogen atoms were assigned isotropic thermal parameters and allowed to ride on their respective parent atoms before the final cycle of least-squares refinement. Oxygen-bound hydrogen atoms were located in difference Fourier maps and and were fixed in these positions with O—H = 0.84 - 0.87 Å and Uiso(H) = 1.2 times Ueq(O).

### Crystal data

**Table:**

[CuBr_2_(C_21_H_15_N_3_)]·0.5H_2_O	*Z* = 4
*M**_r_* = 541.73	*F*(000) = 1064
Triclinic, *P*1	*D*_x_ = 1.879 Mg m^−^^3^
Hall symbol: -P 1	Mo *K*α radiation, λ = 0.71073 Å
*a* = 10.1369 (16) Å	Cell parameters from 5787 reflections
*b* = 10.8131 (17) Å	θ = 2.3–27.5°
*c* = 18.688 (3) Å	µ = 5.33 mm^−^^1^
α = 73.629 (3)°	*T* = 130 K
β = 77.088 (4)°	Prism, blue
γ = 87.567 (5)°	0.35 × 0.25 × 0.10 mm
*V* = 1915.2 (5) Å^3^	

### Data collection

**Table:**

Rigaku Mercury CCD diffractometer	8650 independent reflections
Radiation source: fine-focus sealed tube	7545 reflections with *I* > 2σ(*I*)
graphite	*R*_int_ = 0.021
ω scans	θ_max_ = 27.5°, θ_min_ = 2.3°
Absorption correction: multi-scan (*CrystalClear*; Rigaku, 2002)	*h* = −13→9
*T*_min_ = 0.543, *T*_max_ = 1.000	*k* = −13→14
14948 measured reflections	*l* = −24→24

### Refinement

**Table:**

Refinement on *F*^2^	Primary atom site location: structure-invariant direct methods
Least-squares matrix: full	Secondary atom site location: difference Fourier map
*R*[*F*^2^ > 2σ(*F*^2^)] = 0.032	Hydrogen site location: inferred from neighbouring sites
*wR*(*F*^2^) = 0.079	H-atom parameters constrained
*S* = 1.01	*w* = 1/[σ^2^(*F*_o_^2^) + (0.0455*P*)^2^ + 0.2526*P*] where *P* = (*F*_o_^2^ + 2*F*_c_^2^)/3
8650 reflections	(Δ/σ)_max_ = 0.001
496 parameters	Δρ_max_ = 0.96 e Å^−^^3^
2 restraints	Δρ_min_ = −0.55 e Å^−^^3^

### Special details

**Table:**

Geometry. All esds (except the esd in the dihedral angle between two l.s. planes) are estimated using the full covariance matrix. The cell esds are taken into account individually in the estimation of esds in distances, angles and torsion angles; correlations between esds in cell parameters are only used when they are defined by crystal symmetry. An approximate (isotropic) treatment of cell esds is used for estimating esds involving l.s. planes.
Refinement. Refinement of *F*^2^ against ALL reflections. The weighted *R*-factor wR and goodness of fit *S* are based on *F*^2^, conventional *R*-factors *R* are based on *F*, with *F* set to zero for negative *F*^2^. The threshold expression of *F*^2^ > σ(*F*^2^) is used only for calculating *R*-factors(gt) etc. and is not relevant to the choice of reflections for refinement. *R*-factors based on *F*^2^ are statistically about twice as large as those based on *F*, and *R*- factors based on ALL data will be even larger.

### Fractional atomic coordinates and isotropic or equivalent isotropic displacement parameters (Å^2^)

**Table:**

	*x*	*y*	*z*	*U*_iso_*/*U*_eq_	
Cu1	0.63419 (3)	0.83649 (3)	0.492085 (19)	0.01705 (8)	
Cu2	−0.06492 (3)	0.37614 (3)	0.841659 (19)	0.01615 (8)	
Br1	0.65556 (3)	1.05162 (3)	0.408556 (16)	0.02060 (7)	
Br2	0.84123 (3)	0.72331 (3)	0.421510 (16)	0.02066 (7)	
Br4	−0.28069 (3)	0.49110 (3)	0.885676 (16)	0.02110 (7)	
Br3	−0.14953 (3)	0.18948 (3)	0.817675 (17)	0.02307 (8)	
N1	0.7268 (2)	0.8644 (2)	0.57309 (13)	0.0167 (5)	
N2	0.5554 (2)	0.6933 (2)	0.58000 (13)	0.0153 (5)	
N3	0.4908 (2)	0.7691 (2)	0.45004 (13)	0.0174 (5)	
N4	−0.0272 (2)	0.4935 (2)	0.73239 (13)	0.0173 (5)	
N5	0.0774 (2)	0.4906 (2)	0.84456 (13)	0.0147 (5)	
N6	−0.0299 (2)	0.2903 (2)	0.94745 (13)	0.0162 (5)	
C1	0.8147 (3)	0.9591 (3)	0.56399 (18)	0.0225 (6)	
H1A	0.8395	1.0190	0.5163	0.027*	
C2	0.8705 (3)	0.9714 (3)	0.62327 (18)	0.0232 (6)	
H2A	0.9326	1.0374	0.6153	0.028*	
C3	0.8317 (3)	0.8836 (3)	0.69411 (18)	0.0242 (7)	
H3A	0.8668	0.8906	0.7348	0.029*	
C4	0.7403 (3)	0.7848 (3)	0.70467 (16)	0.0196 (6)	
H4A	0.7128	0.7254	0.7523	0.024*	
C5	0.6909 (3)	0.7763 (3)	0.64275 (16)	0.0162 (6)	
C6	0.5956 (3)	0.6742 (3)	0.64545 (15)	0.0148 (5)	
C7	0.5468 (3)	0.5692 (3)	0.70736 (15)	0.0162 (6)	
H7A	0.5750	0.5568	0.7529	0.019*	
C8	0.4545 (3)	0.4817 (3)	0.70085 (15)	0.0149 (5)	
C9	0.4111 (3)	0.5083 (3)	0.63204 (15)	0.0162 (6)	
H9A	0.3486	0.4535	0.6261	0.019*	
C10	0.4615 (3)	0.6166 (3)	0.57290 (15)	0.0143 (5)	
C11	0.4198 (3)	0.6642 (3)	0.49925 (15)	0.0157 (6)	
C12	0.3101 (3)	0.6132 (3)	0.48241 (16)	0.0202 (6)	
H12A	0.2634	0.5399	0.5160	0.024*	
C13	0.2724 (3)	0.6742 (3)	0.41448 (17)	0.0240 (7)	
H13A	0.1960	0.6454	0.4036	0.029*	
C14	0.3482 (3)	0.7778 (3)	0.36288 (17)	0.0237 (6)	
H14A	0.3269	0.8167	0.3159	0.028*	
C15	0.4572 (3)	0.8224 (3)	0.38314 (16)	0.0225 (6)	
H15A	0.5087	0.8921	0.3487	0.027*	
C16	0.4027 (3)	0.3679 (3)	0.76587 (15)	0.0155 (5)	
C17	0.4695 (3)	0.3253 (3)	0.82606 (17)	0.0219 (6)	
H17A	0.5500	0.3660	0.8236	0.026*	
C18	0.4175 (3)	0.2235 (3)	0.88931 (17)	0.0264 (7)	
H18A	0.4631	0.1965	0.9289	0.032*	
C19	0.2969 (3)	0.1618 (3)	0.89349 (17)	0.0242 (7)	
H19A	0.2600	0.0953	0.9365	0.029*	
C20	0.2329 (3)	0.2004 (3)	0.83316 (18)	0.0229 (6)	
H20A	0.1537	0.1578	0.8352	0.027*	
C21	0.2845 (3)	0.3015 (3)	0.76964 (16)	0.0186 (6)	
H21A	0.2404	0.3254	0.7293	0.022*	
C22	−0.0885 (3)	0.4862 (3)	0.67800 (17)	0.0233 (6)	
H22A	−0.1491	0.4181	0.6879	0.028*	
C23	−0.0655 (3)	0.5767 (3)	0.60657 (16)	0.0217 (6)	
H23A	−0.1100	0.5690	0.5695	0.026*	
C24	0.0240 (3)	0.6776 (3)	0.59162 (16)	0.0203 (6)	
H24A	0.0407	0.7393	0.5443	0.024*	
C25	0.0895 (3)	0.6862 (3)	0.64823 (16)	0.0196 (6)	
H25A	0.1508	0.7534	0.6392	0.024*	
C26	0.0616 (3)	0.5933 (3)	0.71805 (16)	0.0154 (5)	
C27	0.1234 (3)	0.5903 (3)	0.78325 (15)	0.0151 (5)	
C28	0.2202 (3)	0.6760 (3)	0.78426 (16)	0.0167 (6)	
H28A	0.2506	0.7455	0.7417	0.020*	
C29	0.2718 (3)	0.6571 (3)	0.84986 (16)	0.0152 (5)	
C30	0.2229 (3)	0.5513 (3)	0.91277 (16)	0.0168 (6)	
H30A	0.2553	0.5363	0.9572	0.020*	
C31	0.1251 (3)	0.4689 (3)	0.90799 (15)	0.0138 (5)	
C32	0.0636 (3)	0.3526 (3)	0.96800 (15)	0.0148 (5)	
C33	0.0957 (3)	0.3085 (3)	1.03846 (16)	0.0183 (6)	
H33A	0.1587	0.3531	1.0517	0.022*	
C34	0.0331 (3)	0.1969 (3)	1.08938 (17)	0.0226 (6)	
H34A	0.0546	0.1650	1.1370	0.027*	
C35	−0.0619 (3)	0.1330 (3)	1.06902 (17)	0.0240 (7)	
H35A	−0.1048	0.0576	1.1023	0.029*	
C36	−0.0912 (3)	0.1843 (3)	0.99793 (17)	0.0222 (6)	
H36A	−0.1569	0.1431	0.9847	0.027*	
C37	0.3745 (3)	0.7477 (3)	0.85416 (16)	0.0166 (6)	
C38	0.3848 (3)	0.8757 (3)	0.80805 (16)	0.0213 (6)	
H38A	0.3281	0.9038	0.7741	0.026*	
C39	0.4792 (3)	0.9602 (3)	0.81301 (17)	0.0242 (7)	
H39A	0.4841	1.0455	0.7832	0.029*	
C40	0.5666 (3)	0.9183 (3)	0.86238 (18)	0.0252 (7)	
H40A	0.6311	0.9747	0.8649	0.030*	
C41	0.5568 (3)	0.7923 (3)	0.90762 (19)	0.0259 (7)	
H41A	0.6147	0.7640	0.9409	0.031*	
C42	0.4609 (3)	0.7071 (3)	0.90382 (17)	0.0214 (6)	
H42A	0.4547	0.6226	0.9348	0.026*	
O1	−0.0871 (2)	−0.0022 (2)	1.26009 (13)	0.0310 (5)	
H02	−0.0436	−0.0651	1.2466	0.037*	
H01	−0.1332	−0.0381	1.3045	0.037*	

### Atomic displacement parameters (Å^2^)

**Table:**

	*U*^11^	*U*^22^	*U*^33^	*U*^12^	*U*^13^	*U*^23^
Cu1	0.01946 (18)	0.01659 (18)	0.01346 (17)	−0.00567 (13)	−0.00481 (13)	0.00024 (14)
Cu2	0.01875 (18)	0.01616 (18)	0.01344 (16)	−0.00551 (13)	−0.00630 (13)	−0.00099 (14)
Br1	0.02448 (15)	0.01631 (14)	0.01733 (14)	−0.00374 (11)	−0.00329 (11)	0.00075 (11)
Br2	0.02321 (15)	0.02044 (15)	0.01647 (14)	0.00003 (11)	−0.00341 (11)	−0.00287 (11)
Br4	0.02085 (15)	0.02517 (16)	0.02032 (15)	0.00127 (11)	−0.00863 (11)	−0.00809 (12)
Br3	0.02623 (16)	0.01969 (15)	0.02563 (16)	−0.00648 (12)	−0.00917 (12)	−0.00636 (12)
N1	0.0159 (11)	0.0163 (12)	0.0176 (12)	−0.0036 (9)	−0.0051 (9)	−0.0026 (10)
N2	0.0147 (11)	0.0169 (12)	0.0136 (11)	−0.0007 (9)	−0.0026 (9)	−0.0034 (9)
N3	0.0198 (12)	0.0164 (12)	0.0147 (11)	−0.0032 (9)	−0.0050 (9)	−0.0007 (10)
N4	0.0221 (12)	0.0164 (12)	0.0131 (11)	−0.0037 (9)	−0.0053 (9)	−0.0020 (9)
N5	0.0152 (11)	0.0147 (11)	0.0139 (11)	−0.0008 (9)	−0.0048 (9)	−0.0022 (9)
N6	0.0169 (12)	0.0165 (12)	0.0142 (11)	−0.0025 (9)	−0.0047 (9)	−0.0013 (10)
C1	0.0219 (15)	0.0198 (15)	0.0233 (15)	−0.0050 (12)	−0.0053 (12)	−0.0008 (12)
C2	0.0222 (15)	0.0212 (16)	0.0274 (16)	−0.0081 (12)	−0.0071 (12)	−0.0059 (13)
C3	0.0259 (16)	0.0267 (17)	0.0247 (16)	−0.0006 (13)	−0.0120 (12)	−0.0095 (14)
C4	0.0191 (14)	0.0219 (15)	0.0170 (14)	−0.0020 (11)	−0.0050 (11)	−0.0028 (12)
C5	0.0162 (13)	0.0150 (14)	0.0170 (14)	−0.0008 (11)	−0.0020 (10)	−0.0051 (11)
C6	0.0123 (13)	0.0169 (14)	0.0164 (13)	0.0019 (10)	−0.0043 (10)	−0.0061 (11)
C7	0.0171 (13)	0.0210 (15)	0.0117 (13)	−0.0019 (11)	−0.0046 (10)	−0.0048 (11)
C8	0.0146 (13)	0.0144 (13)	0.0139 (13)	−0.0001 (10)	−0.0017 (10)	−0.0023 (11)
C9	0.0162 (13)	0.0167 (14)	0.0162 (13)	−0.0018 (11)	−0.0025 (10)	−0.0059 (11)
C10	0.0144 (13)	0.0158 (13)	0.0137 (13)	−0.0002 (10)	−0.0025 (10)	−0.0061 (11)
C11	0.0184 (14)	0.0148 (14)	0.0135 (13)	0.0007 (11)	−0.0012 (10)	−0.0055 (11)
C12	0.0249 (15)	0.0199 (15)	0.0173 (14)	−0.0076 (12)	−0.0053 (11)	−0.0058 (12)
C13	0.0254 (16)	0.0285 (17)	0.0224 (15)	−0.0058 (13)	−0.0117 (12)	−0.0080 (13)
C14	0.0325 (17)	0.0258 (17)	0.0150 (14)	−0.0029 (13)	−0.0104 (12)	−0.0046 (13)
C15	0.0265 (16)	0.0234 (16)	0.0154 (14)	−0.0053 (12)	−0.0049 (11)	−0.0009 (12)
C16	0.0164 (13)	0.0149 (14)	0.0147 (13)	0.0000 (10)	−0.0035 (10)	−0.0032 (11)
C17	0.0233 (15)	0.0238 (16)	0.0207 (15)	−0.0047 (12)	−0.0100 (12)	−0.0048 (13)
C18	0.0365 (18)	0.0242 (17)	0.0191 (15)	−0.0028 (13)	−0.0123 (13)	−0.0018 (13)
C19	0.0335 (17)	0.0162 (15)	0.0181 (15)	−0.0052 (12)	−0.0030 (12)	0.0018 (12)
C20	0.0201 (15)	0.0183 (15)	0.0279 (16)	−0.0057 (12)	−0.0032 (12)	−0.0033 (13)
C21	0.0192 (14)	0.0176 (14)	0.0183 (14)	−0.0001 (11)	−0.0063 (11)	−0.0023 (12)
C22	0.0296 (16)	0.0237 (16)	0.0191 (15)	−0.0060 (13)	−0.0115 (12)	−0.0041 (13)
C23	0.0276 (16)	0.0242 (16)	0.0162 (14)	−0.0012 (12)	−0.0103 (12)	−0.0059 (12)
C24	0.0213 (14)	0.0239 (16)	0.0125 (13)	0.0015 (12)	−0.0046 (11)	0.0006 (12)
C25	0.0190 (14)	0.0190 (15)	0.0202 (14)	−0.0051 (11)	−0.0051 (11)	−0.0033 (12)
C26	0.0143 (13)	0.0169 (14)	0.0162 (13)	−0.0010 (10)	−0.0023 (10)	−0.0071 (11)
C27	0.0171 (13)	0.0127 (13)	0.0132 (13)	−0.0002 (10)	−0.0011 (10)	−0.0015 (11)
C28	0.0172 (14)	0.0145 (13)	0.0167 (13)	−0.0023 (11)	−0.0034 (10)	−0.0013 (11)
C29	0.0117 (13)	0.0168 (14)	0.0175 (14)	−0.0006 (10)	−0.0035 (10)	−0.0053 (11)
C30	0.0167 (14)	0.0196 (15)	0.0151 (13)	−0.0009 (11)	−0.0062 (10)	−0.0043 (12)
C31	0.0151 (13)	0.0137 (13)	0.0125 (12)	0.0017 (10)	−0.0044 (10)	−0.0027 (11)
C32	0.0139 (13)	0.0150 (14)	0.0152 (13)	−0.0010 (10)	−0.0034 (10)	−0.0037 (11)
C33	0.0202 (14)	0.0187 (14)	0.0165 (14)	−0.0016 (11)	−0.0062 (11)	−0.0037 (12)
C34	0.0286 (16)	0.0223 (16)	0.0159 (14)	0.0020 (12)	−0.0085 (12)	−0.0009 (12)
C35	0.0284 (16)	0.0192 (15)	0.0179 (15)	−0.0080 (12)	−0.0016 (12)	0.0036 (12)
C36	0.0254 (16)	0.0202 (15)	0.0199 (15)	−0.0057 (12)	−0.0071 (12)	−0.0014 (12)
C37	0.0152 (13)	0.0176 (14)	0.0161 (13)	−0.0045 (11)	−0.0001 (10)	−0.0051 (11)
C38	0.0237 (15)	0.0222 (16)	0.0173 (14)	−0.0061 (12)	−0.0053 (11)	−0.0028 (12)
C39	0.0305 (17)	0.0214 (16)	0.0178 (14)	−0.0083 (13)	0.0018 (12)	−0.0050 (12)
C40	0.0208 (15)	0.0306 (18)	0.0249 (16)	−0.0102 (13)	0.0007 (12)	−0.0120 (14)
C41	0.0171 (15)	0.0342 (18)	0.0313 (17)	0.0008 (13)	−0.0095 (12)	−0.0138 (15)
C42	0.0189 (14)	0.0217 (15)	0.0227 (15)	−0.0031 (12)	−0.0057 (11)	−0.0034 (13)
O1	0.0309 (12)	0.0354 (14)	0.0227 (12)	−0.0052 (10)	−0.0039 (9)	−0.0025 (10)

### Geometric parameters (Å, °)

**Table:**

Cu1—N2	1.955 (2)	C16—C21	1.402 (4)
Cu1—N1	2.044 (2)	C17—C18	1.387 (4)
Cu1—N3	2.046 (2)	C17—H17A	0.9300
Cu1—Br1	2.3957 (5)	C18—C19	1.394 (4)
Cu1—Br2	2.6640 (5)	C18—H18A	0.9300
Cu2—N5	1.960 (2)	C19—C20	1.380 (4)
Cu2—N6	2.037 (2)	C19—H19A	0.9300
Cu2—N4	2.039 (2)	C20—C21	1.384 (4)
Cu2—Br3	2.4164 (5)	C20—H20A	0.9300
Cu2—Br4	2.5588 (5)	C21—H21A	0.9300
N1—C1	1.336 (3)	C22—C23	1.392 (4)
N1—C5	1.361 (4)	C22—H22A	0.9300
N2—C6	1.333 (3)	C23—C24	1.375 (4)
N2—C10	1.340 (3)	C23—H23A	0.9300
N3—C15	1.333 (4)	C24—C25	1.393 (4)
N3—C11	1.354 (3)	C24—H24A	0.9300
N4—C22	1.326 (4)	C25—C26	1.383 (4)
N4—C26	1.362 (3)	C25—H25A	0.9300
N5—C31	1.336 (3)	C26—C27	1.482 (4)
N5—C27	1.342 (3)	C27—C28	1.384 (4)
N6—C36	1.334 (4)	C28—C29	1.398 (4)
N6—C32	1.365 (3)	C28—H28A	0.9300
C1—C2	1.391 (4)	C29—C30	1.402 (4)
C1—H1A	0.9300	C29—C37	1.489 (4)
C2—C3	1.378 (4)	C30—C31	1.393 (4)
C2—H2A	0.9300	C30—H30A	0.9300
C3—C4	1.387 (4)	C31—C32	1.479 (4)
C3—H3A	0.9300	C32—C33	1.375 (4)
C4—C5	1.386 (4)	C33—C34	1.383 (4)
C4—H4A	0.9300	C33—H33A	0.9300
C5—C6	1.481 (4)	C34—C35	1.385 (4)
C6—C7	1.387 (4)	C34—H34A	0.9300
C7—C8	1.406 (4)	C35—C36	1.382 (4)
C7—H7A	0.9300	C35—H35A	0.9300
C8—C9	1.401 (4)	C36—H36A	0.9300
C8—C16	1.481 (4)	C37—C42	1.386 (4)
C9—C10	1.387 (4)	C37—C38	1.403 (4)
C9—H9A	0.9300	C38—C39	1.387 (4)
C10—C11	1.476 (4)	C38—H38A	0.9300
C11—C12	1.395 (4)	C39—C40	1.392 (4)
C12—C13	1.384 (4)	C39—H39A	0.9300
C12—H12A	0.9300	C40—C41	1.381 (5)
C13—C14	1.381 (4)	C40—H40A	0.9300
C13—H13A	0.9300	C41—C42	1.394 (4)
C14—C15	1.390 (4)	C41—H41A	0.9300
C14—H14A	0.9300	C42—H42A	0.9300
C15—H15A	0.9300	O1—H02	0.8614
C16—C17	1.399 (4)	O1—H01	0.8485
			
N2—Cu1—N1	79.16 (9)	C17—C16—C21	118.2 (3)
N2—Cu1—N3	79.46 (9)	C17—C16—C8	120.7 (2)
N1—Cu1—N3	157.20 (9)	C21—C16—C8	121.1 (2)
N2—Cu1—Br1	157.21 (7)	C18—C17—C16	121.1 (3)
N1—Cu1—Br1	98.89 (7)	C18—C17—H17A	119.4
N3—Cu1—Br1	97.74 (7)	C16—C17—H17A	119.4
N2—Cu1—Br2	101.40 (7)	C17—C18—C19	119.9 (3)
N1—Cu1—Br2	96.53 (7)	C17—C18—H18A	120.0
N3—Cu1—Br2	95.37 (7)	C19—C18—H18A	120.0
Br1—Cu1—Br2	101.382 (17)	C20—C19—C18	119.3 (3)
N5—Cu2—N6	79.17 (9)	C20—C19—H19A	120.4
N5—Cu2—N4	78.98 (9)	C18—C19—H19A	120.4
N6—Cu2—N4	157.13 (9)	C19—C20—C21	121.1 (3)
N5—Cu2—Br3	154.40 (7)	C19—C20—H20A	119.4
N6—Cu2—Br3	98.60 (7)	C21—C20—H20A	119.4
N4—Cu2—Br3	98.17 (7)	C20—C21—C16	120.3 (3)
N5—Cu2—Br4	102.28 (7)	C20—C21—H21A	119.9
N6—Cu2—Br4	96.87 (7)	C16—C21—H21A	119.9
N4—Cu2—Br4	94.28 (7)	N4—C22—C23	122.4 (3)
Br3—Cu2—Br4	103.308 (18)	N4—C22—H22A	118.8
C1—N1—C5	118.9 (2)	C23—C22—H22A	118.8
C1—N1—Cu1	126.6 (2)	C24—C23—C22	119.0 (3)
C5—N1—Cu1	114.48 (17)	C24—C23—H23A	120.5
C6—N2—C10	121.6 (2)	C22—C23—H23A	120.5
C6—N2—Cu1	119.57 (18)	C23—C24—C25	119.1 (3)
C10—N2—Cu1	118.86 (18)	C23—C24—H24A	120.4
C15—N3—C11	119.2 (2)	C25—C24—H24A	120.4
C15—N3—Cu1	126.59 (19)	C26—C25—C24	118.9 (3)
C11—N3—Cu1	114.09 (18)	C26—C25—H25A	120.5
C22—N4—C26	118.9 (2)	C24—C25—H25A	120.5
C22—N4—Cu2	125.79 (19)	N4—C26—C25	121.6 (2)
C26—N4—Cu2	115.06 (18)	N4—C26—C27	113.7 (2)
C31—N5—C27	121.3 (2)	C25—C26—C27	124.7 (2)
C31—N5—Cu2	119.32 (18)	N5—C27—C28	121.0 (3)
C27—N5—Cu2	119.37 (18)	N5—C27—C26	112.5 (2)
C36—N6—C32	118.2 (2)	C28—C27—C26	126.5 (2)
C36—N6—Cu2	126.87 (19)	C27—C28—C29	119.4 (3)
C32—N6—Cu2	114.85 (18)	C27—C28—H28A	120.3
N1—C1—C2	122.4 (3)	C29—C28—H28A	120.3
N1—C1—H1A	118.8	C28—C29—C30	118.4 (2)
C2—C1—H1A	118.8	C28—C29—C37	121.4 (2)
C3—C2—C1	118.6 (3)	C30—C29—C37	120.2 (2)
C3—C2—H2A	120.7	C31—C30—C29	119.3 (3)
C1—C2—H2A	120.7	C31—C30—H30A	120.4
C2—C3—C4	119.9 (3)	C29—C30—H30A	120.4
C2—C3—H3A	120.1	N5—C31—C30	120.6 (2)
C4—C3—H3A	120.1	N5—C31—C32	112.7 (2)
C5—C4—C3	118.6 (3)	C30—C31—C32	126.6 (2)
C5—C4—H4A	120.7	N6—C32—C33	121.8 (2)
C3—C4—H4A	120.7	N6—C32—C31	113.8 (2)
N1—C5—C4	121.6 (2)	C33—C32—C31	124.4 (2)
N1—C5—C6	114.1 (2)	C32—C33—C34	119.1 (3)
C4—C5—C6	124.3 (3)	C32—C33—H33A	120.5
N2—C6—C7	120.7 (2)	C34—C33—H33A	120.5
N2—C6—C5	112.6 (2)	C33—C34—C35	119.5 (3)
C7—C6—C5	126.7 (2)	C33—C34—H34A	120.2
C6—C7—C8	119.6 (2)	C35—C34—H34A	120.2
C6—C7—H7A	120.2	C36—C35—C34	118.2 (3)
C8—C7—H7A	120.2	C36—C35—H35A	120.9
C9—C8—C7	117.7 (2)	C34—C35—H35A	120.9
C9—C8—C16	121.8 (2)	N6—C36—C35	123.1 (3)
C7—C8—C16	120.5 (2)	N6—C36—H36A	118.5
C10—C9—C8	119.8 (2)	C35—C36—H36A	118.5
C10—C9—H9A	120.1	C42—C37—C38	119.1 (3)
C8—C9—H9A	120.1	C42—C37—C29	120.6 (3)
N2—C10—C9	120.4 (2)	C38—C37—C29	120.3 (3)
N2—C10—C11	112.6 (2)	C39—C38—C37	120.2 (3)
C9—C10—C11	126.9 (2)	C39—C38—H38A	119.9
N3—C11—C12	121.4 (3)	C37—C38—H38A	119.9
N3—C11—C10	114.6 (2)	C38—C39—C40	120.4 (3)
C12—C11—C10	123.8 (2)	C38—C39—H39A	119.8
C13—C12—C11	118.4 (3)	C40—C39—H39A	119.8
C13—C12—H12A	120.8	C41—C40—C39	119.5 (3)
C11—C12—H12A	120.8	C41—C40—H40A	120.3
C14—C13—C12	120.0 (3)	C39—C40—H40A	120.3
C14—C13—H13A	120.0	C40—C41—C42	120.5 (3)
C12—C13—H13A	120.0	C40—C41—H41A	119.7
C13—C14—C15	118.2 (3)	C42—C41—H41A	119.7
C13—C14—H14A	120.9	C37—C42—C41	120.4 (3)
C15—C14—H14A	120.9	C37—C42—H42A	119.8
N3—C15—C14	122.5 (3)	C41—C42—H42A	119.8
N3—C15—H15A	118.7	H02—O1—H01	103.3
C14—C15—H15A	118.7		
			
N2—Cu1—N1—C1	−179.0 (3)	Cu1—N3—C11—C10	−1.4 (3)
N3—Cu1—N1—C1	−158.4 (3)	N2—C10—C11—N3	5.2 (3)
Br1—Cu1—N1—C1	−22.1 (3)	C9—C10—C11—N3	−176.8 (3)
Br2—Cu1—N1—C1	80.6 (2)	N2—C10—C11—C12	−170.2 (2)
N2—Cu1—N1—C5	0.08 (19)	C9—C10—C11—C12	7.8 (4)
N3—Cu1—N1—C5	20.7 (4)	N3—C11—C12—C13	−1.6 (4)
Br1—Cu1—N1—C5	157.03 (18)	C10—C11—C12—C13	173.5 (3)
Br2—Cu1—N1—C5	−100.32 (19)	C11—C12—C13—C14	4.2 (5)
N1—Cu1—N2—C6	−2.8 (2)	C12—C13—C14—C15	−3.5 (5)
N3—Cu1—N2—C6	−174.9 (2)	C11—N3—C15—C14	2.7 (4)
Br1—Cu1—N2—C6	−90.0 (3)	Cu1—N3—C15—C14	−172.7 (2)
Br2—Cu1—N2—C6	91.7 (2)	C13—C14—C15—N3	0.0 (5)
N1—Cu1—N2—C10	176.9 (2)	C9—C8—C16—C17	164.4 (3)
N3—Cu1—N2—C10	4.9 (2)	C7—C8—C16—C17	−17.5 (4)
Br1—Cu1—N2—C10	89.8 (3)	C9—C8—C16—C21	−16.9 (4)
Br2—Cu1—N2—C10	−88.5 (2)	C7—C8—C16—C21	161.2 (3)
N2—Cu1—N3—C15	174.0 (3)	C21—C16—C17—C18	−2.6 (4)
N1—Cu1—N3—C15	153.4 (3)	C8—C16—C17—C18	176.2 (3)
Br1—Cu1—N3—C15	16.9 (3)	C16—C17—C18—C19	0.1 (5)
Br2—Cu1—N3—C15	−85.4 (2)	C17—C18—C19—C20	2.1 (5)
N2—Cu1—N3—C11	−1.62 (19)	C18—C19—C20—C21	−1.7 (5)
N1—Cu1—N3—C11	−22.2 (4)	C19—C20—C21—C16	−0.8 (5)
Br1—Cu1—N3—C11	−158.71 (18)	C17—C16—C21—C20	2.9 (4)
Br2—Cu1—N3—C11	99.01 (19)	C8—C16—C21—C20	−175.9 (3)
N5—Cu2—N4—C22	180.0 (3)	C26—N4—C22—C23	0.0 (4)
N6—Cu2—N4—C22	162.6 (3)	Cu2—N4—C22—C23	174.3 (2)
Br3—Cu2—N4—C22	25.8 (3)	N4—C22—C23—C24	0.0 (5)
Br4—Cu2—N4—C22	−78.3 (3)	C22—C23—C24—C25	0.2 (4)
N5—Cu2—N4—C26	−5.53 (19)	C23—C24—C25—C26	−0.4 (4)
N6—Cu2—N4—C26	−22.9 (4)	C22—N4—C26—C25	−0.3 (4)
Br3—Cu2—N4—C26	−159.71 (18)	Cu2—N4—C26—C25	−175.2 (2)
Br4—Cu2—N4—C26	96.17 (19)	C22—N4—C26—C27	−179.9 (3)
N6—Cu2—N5—C31	−2.8 (2)	Cu2—N4—C26—C27	5.2 (3)
N4—Cu2—N5—C31	−176.0 (2)	C24—C25—C26—N4	0.5 (4)
Br3—Cu2—N5—C31	−90.1 (2)	C24—C25—C26—C27	−180.0 (3)
Br4—Cu2—N5—C31	92.0 (2)	C31—N5—C27—C28	−1.4 (4)
N6—Cu2—N5—C27	178.3 (2)	Cu2—N5—C27—C28	177.5 (2)
N4—Cu2—N5—C27	5.1 (2)	C31—N5—C27—C26	177.4 (2)
Br3—Cu2—N5—C27	90.9 (3)	Cu2—N5—C27—C26	−3.6 (3)
Br4—Cu2—N5—C27	−87.0 (2)	N4—C26—C27—N5	−1.2 (3)
N5—Cu2—N6—C36	−179.8 (3)	C25—C26—C27—N5	179.2 (3)
N4—Cu2—N6—C36	−162.4 (2)	N4—C26—C27—C28	177.5 (3)
Br3—Cu2—N6—C36	−25.7 (2)	C25—C26—C27—C28	−2.1 (5)
Br4—Cu2—N6—C36	78.9 (2)	N5—C27—C28—C29	0.9 (4)
N5—Cu2—N6—C32	2.47 (19)	C26—C27—C28—C29	−177.8 (3)
N4—Cu2—N6—C32	19.8 (4)	C27—C28—C29—C30	−0.2 (4)
Br3—Cu2—N6—C32	156.58 (18)	C27—C28—C29—C37	−179.1 (2)
Br4—Cu2—N6—C32	−98.78 (18)	C28—C29—C30—C31	0.0 (4)
C5—N1—C1—C2	−0.3 (4)	C37—C29—C30—C31	178.9 (2)
Cu1—N1—C1—C2	178.7 (2)	C27—N5—C31—C30	1.2 (4)
N1—C1—C2—C3	−1.0 (5)	Cu2—N5—C31—C30	−177.7 (2)
C1—C2—C3—C4	0.9 (5)	C27—N5—C31—C32	−178.6 (2)
C2—C3—C4—C5	0.6 (5)	Cu2—N5—C31—C32	2.5 (3)
C1—N1—C5—C4	1.8 (4)	C29—C30—C31—N5	−0.5 (4)
Cu1—N1—C5—C4	−177.3 (2)	C29—C30—C31—C32	179.2 (3)
C1—N1—C5—C6	−178.5 (2)	C36—N6—C32—C33	0.3 (4)
Cu1—N1—C5—C6	2.3 (3)	Cu2—N6—C32—C33	178.3 (2)
C3—C4—C5—N1	−2.0 (4)	C36—N6—C32—C31	−179.8 (2)
C3—C4—C5—C6	178.5 (3)	Cu2—N6—C32—C31	−1.9 (3)
C10—N2—C6—C7	3.8 (4)	N5—C31—C32—N6	−0.3 (3)
Cu1—N2—C6—C7	−176.5 (2)	C30—C31—C32—N6	179.9 (3)
C10—N2—C6—C5	−175.0 (2)	N5—C31—C32—C33	179.6 (3)
Cu1—N2—C6—C5	4.8 (3)	C30—C31—C32—C33	−0.2 (5)
N1—C5—C6—N2	−4.5 (3)	N6—C32—C33—C34	1.0 (4)
C4—C5—C6—N2	175.1 (3)	C31—C32—C33—C34	−178.8 (3)
N1—C5—C6—C7	176.9 (3)	C32—C33—C34—C35	−1.0 (4)
C4—C5—C6—C7	−3.5 (5)	C33—C34—C35—C36	−0.4 (5)
N2—C6—C7—C8	0.3 (4)	C32—N6—C36—C35	−1.8 (4)
C5—C6—C7—C8	178.9 (3)	Cu2—N6—C36—C35	−179.4 (2)
C6—C7—C8—C9	−2.7 (4)	C34—C35—C36—N6	1.8 (5)
C6—C7—C8—C16	179.1 (2)	C28—C29—C37—C42	−155.7 (3)
C7—C8—C9—C10	1.3 (4)	C30—C29—C37—C42	25.4 (4)
C16—C8—C9—C10	179.5 (2)	C28—C29—C37—C38	24.5 (4)
C6—N2—C10—C9	−5.3 (4)	C30—C29—C37—C38	−154.4 (3)
Cu1—N2—C10—C9	175.0 (2)	C42—C37—C38—C39	−0.7 (4)
C6—N2—C10—C11	172.9 (2)	C29—C37—C38—C39	179.1 (3)
Cu1—N2—C10—C11	−6.9 (3)	C37—C38—C39—C40	1.5 (4)
C8—C9—C10—N2	2.6 (4)	C38—C39—C40—C41	−1.3 (4)
C8—C9—C10—C11	−175.2 (3)	C39—C40—C41—C42	0.4 (5)
C15—N3—C11—C12	−1.9 (4)	C38—C37—C42—C41	−0.2 (4)
Cu1—N3—C11—C12	174.1 (2)	C29—C37—C42—C41	180.0 (3)
C15—N3—C11—C10	−177.4 (3)	C40—C41—C42—C37	0.4 (4)

### Hydrogen-bond geometry (Å, °)

**Table:**

*D*—H···*A*	*D*—H	H···*A*	*D*···*A*	*D*—H···*A*
O1—H01···Br1^i^	0.85	2.89	3.520 (2)	133
O1—H01···Br2^i^	0.85	2.85	3.549 (2)	141
O1—H02···Br3^ii^	0.86	2.59	3.414 (2)	160

Symmetry codes: (i) *x*−1, *y*−1, *z*+1; (ii) −*x*, −*y*, −*z*+2.
